# Utilization of Nonlinear Parametric Resonance in Micro Sensor Probes to Enhance Atomic Force Microscope Resolution

**DOI:** 10.3390/s26154791

**Published:** 2026-07-28

**Authors:** Jonathan Ehrmann, Oliver Radler, Thomas Sattel

**Affiliations:** Mechatronics Group, Technische Universität Ilmenau, Max-Planck-Ring 12, 98693 Ilmenau, Germanythomas.sattel@tu-ilmenau.de (T.S.)

**Keywords:** Atomic Force Microscopy, resolution, sensitivity, thermomechanical noise, nonlinear parametric resonance

## Abstract

Atomic Force Microscopy (AFM) uses oscillating cantilever-shaped microprobes to measure nanometer-scaled sample topography. Spatial resolution of AM-AFM is determined by the dynamics of the cantilever-sample system including thermomechanical noise of the cantilever. Conventional AFM systems drive the cantilever harmonically in resonance, where resolution can only be improved by changing system and process parameters. We take a different approach keeping cantilever, sample, and control method (AM-AFM) unchanged. Instead, we operate the AFM cantilever in nonlinear parametric resonance. The corresponding excitation scheme is produced via electronic feedback. Because of the artificial source of the nonlinear parametric excitation, we implement arbitrary system behavior and prove the theoretical findings experimentally. We analyze the influence of excitation parameters on dynamic system behavior including an explanation of the nonlinear limitation mechanism of the cantilever amplitude in parametric instability and amplitude reduction mechanism during approach of the cantilever to the sample. We find a mechanism which could reduce tip damage. The dependence of the cantilever’s thermomechanical noise on parametric excitation is investigated. Our analysis yields the resolution of parametric resonance AFM and enables systematic selection of parametric excitation parameters to improve resolution of existing AFM systems. We provide evidence that responsivity is enhanced by 30%, thermomechanical noise by a factor of 4.5, and resolution by a factor of 5.7 for identical systems when parametric resonance AFM is used.

## 1. Introduction

Atomic Force Microscopy (AFM) is a surface analysis technique that uses microcantilevers to investigate the topography of a sample surface [1]. Mostly, dynamic AFM (dAFM) with amplitude modulation is used, where the cantilever oscillates over the sample surface and experiences tip–sample interaction forces that are nonlinearly dependent on the distance between the cantilever tip and sample surface. From a dynamic point of view, dAFM uses a forced excitation of the cantilever in the vicinity of its first bending resonance frequency [2]. Approaching the tip of the cantilever and the sample surface to each other, the tip–sample interaction force changes, and with that the displacement amplitude of the cantilever. By bringing both quantities, the displacement amplitude (referred to as amplitude) and the quasistatic tip–sample distance (kinematics explained in Materials and Methods), together, the so-called amplitude–distance curve (approach curve) is gained. This is an important characteristic of the cantilever-sample system (referred to as system) and contributes to the spatial resolution in the vertical direction of the AFM system [3,4].

As in many measuring applications, resolution is also the most important characteristic in AFM and resonant sensors in general. Resolution is defined as the smallest detectable change of a measured quantity which is limited by noise [5] (p. 177). As a synonym for resolution, the term sensitivity is often used. Before, we applied this definition to the context of amplitude-modulated AFM and showed the dependence of sensitivity on different system parameters [4]. For this, we introduced several characteristics as generally stated for resonant sensors [5] (p. 175 ff.) by looking at the cantilever-sample system as a force sensor. First, the amplitude responsivity $R_{\hat{q}}$ was defined as the derivative of the function $\hat{q}\left(\kappa_{\mathrm{ts}}\right)$, where $\hat{q}$ is the cantilever amplitude and $\kappa_{\mathrm{ts}}$ a normalized measure of the strength of the tip–sample interaction force (explained later in this work). So, amplitude responsivity is defined as [4] (Equation (19))

(1) $$R_{\hat{q}}=\frac{\partial \hat{q}}{\partial \kappa_{\mathrm{ts}}},$$

and indicated how strongly the cantilever responds to tip–sample interaction. By including the noise that limits measurement, we come to the spatial resolution in vertical direction, in this work called amplitude sensitivity, which is defined as [4], [5] (Equation (5))

(2) $$S_{\hat{q}}:=\frac{\delta {\hat{q}}_{\mathrm{th}}}{R_{\hat{q}}},$$

where $\delta {\hat{q}}_{\mathrm{th}}$ is the limiting noise of thermomechanical nature, in this context also called amplitude noise [2]. Equation (2) shows that a high amplitude responsivity and low amplitude noise lead to better amplitude sensitivity, which is expressed by lower values of $S_{\hat{q}}$.

To enhance sensitivity, either the amplitude responsivity $R_{\hat{q}}$ or the amplitude noise $\delta {\hat{q}}_{\mathrm{th}}$ must be improved. Amplitude responsivity, for example, can be improved by steepening the resonance curve of the cantilever [2,4]. The classical approach to this is to modify the quality factor *Q* of the cantilever by transitioning to other environments, such as vacuum [6], or by employing feedback control methods such as Q-Control [7]. This leads to disadvantages, such as a reduction in possible scanning speed [8] and an increase of amplitude noise [2,9]. Both amplitude responsivity and amplitude noise can also be enhanced in other ways. All of them have in common that the parameters of the system need to be changed [4].

In this work, we take another approach and keep the system regarding the AM-AFM process constant, meaning that we do not change any cantilever or process parameters. In addition, the control method (amplitude modulation) is retained. Instead, we change the way the cantilever is excited by exploiting nonlinear dynamics. More specifically, we make use of parametric oscillators driven in parametric resonance, where the oscillation amplitude is limited by a nonlinearity. The parametric excitation and the nonlinearity are added to the system on purpose and artificially by a feedback loop. Therefore, they can be adjusted, and it is possible to flexibly design the system behavior and achieve better sensitivity compared to the identical system with forced excitation. The idea is to steepen the resonance curve without modifying the quality factor (damping) of the system or other parameters to overcome the mentioned disadvantages.

In general, parametric oscillators are systems in which a system parameter, for example, the stiffness, changes periodically. Such systems can be described by the famous Mathieu equation [10]

(3) $$q^{\prime \prime }+\left(\alpha +\beta \cos\tau \right)q=0,$$

with the generalized displacement *q*, their second derivative to the dimensionless time $q^{\prime \prime }$, excitation amplitude β and frequency ratio $\alpha ={\left(\omega_{0}/\Omega \right)}^{2}$ with eigenfrequency $\omega_{0}$, and excitation frequency Ω of the oscillator. By setting $\alpha =n^{2}/4$ with n=1,2… the oscillator equilibrium becomes unstable, meaning that a perturbation of the equilibrium leads to unbounded oscillations and the amplitude diverges. More detailed, in the β-α-plane (Strutt–Ince diagram) are whole regions with unstable behavior, the so-called instability tongues. In the case of instability, the oscillator is in a parametric resonance. In order to use parametric resonances, nonlinearities are needed that limit the amplitude of the oscillator and enable stationary oscillations. In case of a damped system, the instability tongues move up along the β-axis of the Strutt–Ince diagram, meaning that parametric resonance is entered for values $\beta \geq \beta_{\mathrm{th}}\geq 0$, where $\beta_{\mathrm{th}}$ marks the threshold for parametric resonance [10].

In general, these kinds of system are widely investigated. Also, in the context of AFM, a series of works has been published that used parametric excitation of the cantilever [8,11,12]. In particular, these works modified a commercial AFM system to integrate parametric excitation by building a feedback circuit with the displacement signal of the cantilever. Nevertheless, the nonlinearity in these works is inherent and therefore not adjustable in any sense. Further, there was no dynamic analysis or consideration of noise to find the possible sensitivity.

Also, in other works with a more general MEMS application, parametric resonances are used [13]. In all of the considered works, the nonlinearity in the system is of cubic type and is inherently set by the system [8,11,12,14,15,16,17,18,19,20,21,22,23], for example, due to mechanic or electrostatic nonlinearity. The point where the works differ from each other is the mathematical structure of the system equations. Some of the systems are described by the nonlinear Mathieu equation [19,20,21,22], meaning that there is also a cubic stiffness which is modulated,

(4) $$q^{\prime \prime }+2Dq^{\prime }+\left(1+G\cos\eta \tau \right)\;q+\left(\gamma_{1}+\gamma_{2}\cos\eta \tau \right)\;q^{3}=0,$$

with damping ratio *D*, dimensionless excitation frequency $\eta =\Omega /\omega_{0}$, excitation amplitude *G*, static cubic stiffness $\gamma_{1}$, and nonlinearity factor $\gamma_{2}$. Others are of the Mathieu-Duffing equation type, which means that the Mathieu equation is combined with a static cubic stiffness γ [14,15,16,17,18,23],

(5) $$q^{\prime \prime }+2Dq^{\prime }+\left(1+G\cos\eta \tau \right)\;q+\gamma q^{3}=0.$$

Both types of systems, (4) and (5), have in common that they lead to asymmetric amplitude response curves (cantilever amplitude as a function of excitation frequency, $\hat{q}\left(\eta \right)$) due to the static cubic stiffness and therefore jump phenomena and unstable branches, see, i.e., [15,24]. In contrast, in Prakash et al. symmetric amplitude response curves are gained [12]. The cause for that is the origin of the nonlinearity, which is part of their feedback circuit—a more detailed part of the displacement sensor—instead of the MEM system itself as in other works. This fact is debated later in this work.

Analysis regarding sensitivity enhancement due to parametric excitation is present in only a few works and makes use of bifurcation detection to enhance the sensitivity, which means the detection of the transition between parametric resonance and trivial equilibrium [15,16,17,19,25]. These devices are used as mass sensors and achieve high frequency resolutions (Requa and Turner achieved $d\omega_{0}/\omega_{0}\approx 10^{-7}$, [17]). However, in our view, they do not allow for continuous AFM operation. Works that continuously operate amplitude-modulated AFM with parametric excitation do not analyze the influence of noise on the system and the resulting sensitivity [8,11,12]. Here, we see a significant deficit.

In general, the thermomechanical noise of the system can be modeled as a so-called thermal noise force $F_{\mathrm{th}}$, which represents a stationary stochastic process [26]. This force acts dynamically like a harmonic forcing term and is therefore on the right side of the system equation. For this reason, this type of noise is called additive noise, which is independent of the oscillator state variables, in contrast to multiplicative noise as another type which depends on a state variable of the oscillator [27] (p. 9). The power spectral density of $F_{\mathrm{th}}$ is

(6) $$S_{\mathrm{FF}}\left(\Omega \right)=\frac{4k_{B}Tm\omega_{0}}{Q}$$

and depends on the parameters of the oscillator, *Q* and modal mass *m*, as well as the Boltzmann constant $k_{B}$ and the temperature *T* [6] (Equation (14)), [9].

In order to analyze the influence of this thermal noise force on the parametrically excited AFM system and to further determine the possible sensitivity, a general review of parametric oscillators under the influence of (additive) noise is necessary. First, if the amplitude fluctuations are small compared to the deterministic oscillator amplitude ${\hat{q}}_{\det}$, even for nonlinear systems that are operated in parametric resonance, the fluctuations of their amplitude are found to follow a Gaussian distribution with a mean value that corresponds to the deterministic amplitude (without noise) of the system [28]. This enables the analysis of the system using the statistical properties mean μ and variance $\sigma^{2}$, so the variance of the fluctuations corresponds to the mean squared value of the amplitude noise and its square root, the standard deviation, is the RMS value of the amplitude noise, $\sqrt{\sigma_{\hat{q}}^{2}}=\sigma_{\hat{q}}=\delta \hat{q}$ [29] (p. 27), which is needed to calculate the sensitivity (2).

There are analytical works on linear systems with and without damping that have found that the statistical properties of a parametric oscillator, more detailed than the variance of the oscillator amplitude $\sigma_{\hat{q}}^{2}$, behave like the properties of the deterministic system (amplitude $\hat{q}$) predicted by Floquet theory. This means that $\sigma_{\hat{q}}^{2}$ in parametric resonance grows exponentially and unboundedly as the deterministic amplitude ${\hat{q}}_{\det}$ of the oscillator [30,31].

If the system is nonlinear, in order to limit the amplitude in parametric resonance and achieve a stationary oscillation, there is a study that analyzes a parametric oscillator with additive and multiplicative noise [32]. The result is that the variance of the amplitude of the oscillator diverges if the parametric excitation amplitude is near the threshold for parametric resonance. Upon entering resonance and further increasing the excitation amplitude, the variance shrinks again and, in the case of additive noise only, converges to zero. Again, the mean value of the fluctuating amplitude corresponds to the value of the deterministic amplitude.

So, if the thermomechanical noise is considered to be small, the findings of the listed works can be applied to an AFM system model with parametric excitation to calculate the theoretical sensitivity. This assumption is proofed and validated later in this work.

To conclude, there is potential to enhance amplitude-modulated AFM sensitivity using parametric excitation. Previous works that proved sensitivity enhancement on AFM cantilevers using parametric excitation rely on bifurcation detection, which would require an entirely different operation. Here, we apply parametric excitation on amplitude-modulated AFM with the goal of keeping the entire system untouched except the excitation of the cantilever. Existing works implementing parametric excitation via electronic feedback are still limited to inherent nonlinearities. In this work, the nonlinear behavior is part of the design process. Investigations on the influence of thermomechanical noise on the parametrically excited cantilever and its effect on the sensitivity of the AFM system are outstanding. Furthermore, more sophisticated systems, meaning a systematic design of the nonlinear behavior, have not been investigated yet.

The structure of this paper is as follows. First, possible excitation schemes, that is different mathematical equations for parametric excitation, should be compared to find a suitable excitation scheme that follows the overall goal of enhancing the sensitivity of AFM. Second, the general dynamic behavior of the selected excitation scheme is analyzed to gain an understanding of parameter influences. As the next step, the impact of thermomechanical noise on the amplitude fluctuations of the parametrically excited system is investigated. With these findings, the theoretical sensitivity of the parametric resonance AFM is calculated along with several experimental results to validate the numerical findings. For this, we apply the previously presented definition of sensitivity for the forced excited AFM as well as our numerical and experimental procedures to calculate and measure the approach curves to the novel system with parametric excitation. In the end, the parametric excitation mode is compared to the conventional forced excitation with the result that the novel excitation mode outperforms the conventional mode with respect to sensitivity.

## 2. Materials and Methods

### 2.1. Kinematics

**Figure 1:**
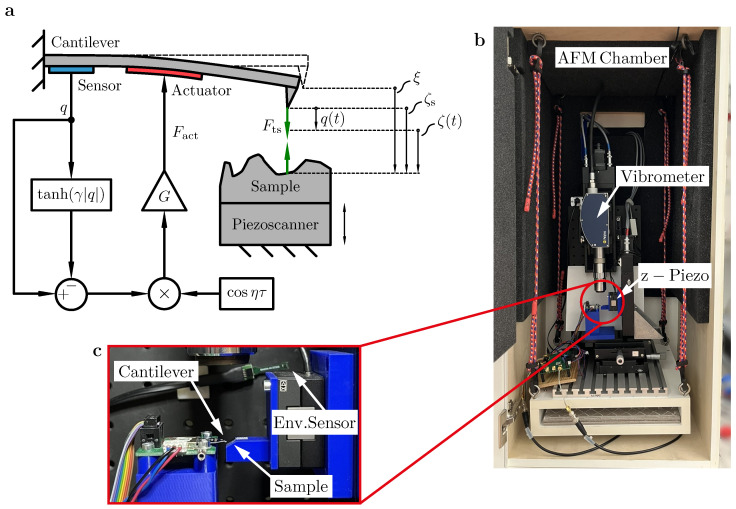
(**a**) Schematic experimental setup showing the kinematics of the cantilever-sample system with the approach distance ξ, the quasistatic tip displacement $\zeta_{s}$ and the tip displacement ζ(t). Equation (11) is implemented experimentally by an electronic feedback circuit. (**b**,**c**) Self-built AFM chamber containing the experimental setup. Suspension and foam serve as passive isolation from acoustic and environmental influences. Feedback electronics are located outside the chamber.

To clearly define the coordinates used, Figure 1a depicts the kinematics of the cantilever–sample system as defined before [4]. The approach distance ξ denotes how close the cantilever is brought to the sample. At this distance, the tip–sample interaction force is already non-negligible. Consequently, a quasistatic tip–sample distance $\zeta_{s}$ arises from the quasistatic displacement of the cantilever. Once the cantilever begins to oscillate, $\zeta_{s}$ represents the equilibrium separation between tip and sample. The instantaneous tip–sample distance ζ incorporates the current cantilever displacement *q* due to oscillation and is given by $\zeta =\zeta_{s}-q$.

### 2.2. Numerical Simulations

The procedure described here is based on the simulations in [4]. The system equation that is later developed in Section 3.1 originates from its unscaled version

(7) $$\tilde{m}\ddot{\tilde{q}}+\tilde{d}\dot{\tilde{q}}+\left[\tilde{k}+\tilde{G}\left(1-\tanh\left(\tilde{\gamma }\left|\tilde{q}\right|\right)\right)\cos\tilde{\Omega }t\right]\tilde{q}={\tilde{F}}_{\mathrm{ts}}$$

where $\tilde{□}$ marks unscaled quantities with physical units, mass *m*, damping coefficient *d*, and stiffness *k*. To get better numerical results, Equation (7) is scaled using a dimensionless time $\tau =\omega_{0}t$ with the eigenfrequency (excluding tip–sample interaction) of the cantilever $\omega_{0}$ and by introducing the damping ratio $D=\frac{1}{2\omega_{0}}\frac{d}{m}\approx \frac{1}{2D}$. All parameters with length units are scaled to $q_{\mathrm{ref}}=1$ nm, mass units are scaled to $m_{\mathrm{ref}}=1$ ng, and temperatures are scaled to $T_{\mathrm{ref}}=300$ K. So, (7) transforms into

(8) $$q^{\prime \prime }+2Dq^{\prime }+q\left[1+G\left(1-\tanh\left(\gamma |q|\right)\right)\cos\eta \tau \right]=0,$$

which is equal to the equation developed later in Section 3.1. Although the results of (8) are dimensionless, we use the unit nanometer ($10^{-9}$ m) for length quantities in all plots for a more intuitive understanding, since the values themselves are identical. All parameters describing the cantilever are obtained from experiments, and sample properties are obtained from the literature. Both are listed in Table 1.

The numerical simulations are performed in Matlab and make use of time integration solvers to compute a periodic orbit as initial guess for a pseudo-arclength continuation solver (Continuation Core Toolbox [33]) to follow this initial guess and calculate the presented bifurcation diagrams. During the continuation, a corresponding value of $\kappa_{\mathrm{ts}}$ is calculated at each computational step, as well as the amplitude using an FFT algorithm. Also, the stability of each computed periodic orbit is analyzed as part of the Continuation Core Toolbox that counts unstable Floquet multipliers to determine Lyapunov stability [33].

**Table 1:** Parameters used in experiment and computation.

Parameter	Symbol	Value	Unit
Resonance Frequency	$f_{0}=\omega_{0}/\left(2\pi \right)$	62.291	kHz
Modal Stiffness	*k*	28	N/m
Quality Factor	Q≈1/(2D)	374	
Tip Radius	*R*	17	nm
Hamaker Constant [34]	*A*	$15\cdot 10^{-20}$	J
Intermolecular Distance [35]	$a_{0}$	0.4	nm
Temperature	*T*	300	K
Boltzmann Constant	$k_{B}$	$1.38\cdot 10^{-23}$	J/K
Measurement Bandwidth	*B*	1	kHz
Scaling Parameter Length	$q_{\mathrm{ref}}$	1	nm
Scaling Parameter Mass	$m_{\mathrm{ref}}$	1	ng
Scaling Parameter Temperature	$T_{\mathrm{ref}}$	300	K

### 2.3. Noise Simulations

The numerical simulations for the investigation of the thermomechanical noise behavior are performed in Matlab Simulink. The implementation of the thermal noise force in (14) is realized by its power spectral density (6) using the Matlab Simulink block Band-Limited White Noise [36]. To obtain real stochastic noise, the Matlab random number generator underlying the Simulink block used is randomized using rng(‘shuffle’). The bandwidth of the generated white noise is limited to $100\;\omega_{0}$ of the cantilever. The system Equation (14) is also implemented in Simulink. The calculated displacement signal *q* is filtered with a first order Butterworth bandpass to simulate a real measurement setup where a lock-in amplifier filters the displacement signal of the cantilever in a band $\omega \in \left[\omega_{0}-B/2,\;\omega_{0}+B/2\right]$ with the bandwidth *B* according to Table 1. The implemented model is time integrated for $10^{6}$ cantilever oscillation periods with automatic Simulink solver settings. The result is a cantilever displacement signal over time with fluctuating amplitude as an effect of the thermomechanical noise at the system input.

The post-processing contains several steps to statistically analyze the computed amplitude fluctuations of the displacement signal. First, the transients are cut off to obtain a purely stationary signal. Second, the signal is divided into *N* measurement windows, each with the size of an oscillation period. Next, correspondingly to the *N* measurement windows, *N* amplitude values are calculated using ${\hat{q}}_{i}=\left(\max(q)-\min(q)\right)/2,\;i=1,\ldots ,N$ per measurement window. This is sufficient for the determination of the amplitude values because the implemented bandpass filter already filters out frequency components far from $\omega_{0}$ and thus prevents high-frequency noise from biasing the statistical evaluation. Finally, the statistical properties, namely the mean $\bar{\hat{q}}$ and standard deviation $\sigma_{\hat{q}}$, of the amplitude vector are computed.

The described procedure is performed for all parameter sets presented.

### 2.4. Experiments

The experimental setup is shown in Figure 1b,c. For all experiments, an active cantilever (active probe) with an integrated sensor and actuator from nano analytik GmbH, Ilmenau, Germany is used (length: ≈350 μm, width: ≈55 μm, height: ≈6.5 μm). The relevant parameters of the cantilever are listed in Table 1. The methods for determining the cantilever parameters as well as the general measurement setup is reported in [4] (Figure 9), namely frequency sweeps for the first bending resonance frequency and quality factor and thermal noise spectra for the modal stiffness. Measurement devices among others are a laser vibrometer OFV-5000 by Polytec, Waldbronn, Germany (displacement measurement resolution 0.015 nm) and a lock-in amplifier Moku:Go by Liquid Instruments, San Diego, USA with filter cut-off frequency 5 kHz, both for measuring the cantilever’s motion. In addition to this previously used setup, a self-built AFM chamber is used to isolate the setup from acoustic and environmental influences. The temperature in the chamber during all measurements is 30 °C and the relative humidity is 26% (measured by environmental sensor, see Figure 1c). The chamber contains normal air.

The parametric excitation of the cantilever is realized using a Moku:Go device with implemented FPGA code that implements a signal processing chain that follows Equation (11), which results in an actuator force

(9) $$F_{\mathrm{act}}=-q\;G\left(1-\tanh\left(\gamma |q|\right)\right)\cos\eta \tau ,$$

see also Figure 1. The parametric excitation parameters *G* and γ are implemented as digital input registers C1 and C3. The cosine signal is generated by an external frequency generator, Handyscope HS5 from TiePie engineering, Sneek, The Netherlands.

The presented amplitude response curves are obtained by measuring a stepped frequency sweep. At each frequency step, we wait for the cantilever oscillation to reach the stationary oscillation and measure 5000 oscillation periods. The measured data is post-processed using an FFT algorithm to calculate the amplitude response curve.

The influence of the excitation parameters on the cantilever amplitude noise is measured using the same setup as for the amplitude response curve. In contrast to that, 100,000 periods of the cantilever oscillation are measured for every parameter step. The post-processing is equal to the noise simulations described previously.

The actual measurement of the amplitude–distance curves was reported before [4]: To approach the cantilever and the sample to each other, a piezo scanner PX 100 from piezosysteme jena, Jena, Germany is used (travel range: 100 μm), which is controlled by our self-developed electronics and which was previously calibrated to ensure linear motion behavior [4]. The piezo position signal is obtained by measuring the voltage applied to the piezo and converting it to a position signal by applying the calibration curve. This signal contains high-frequency noise, which can be filtered because the mechanical transfer behavior of the piezo scanner prevents the voltage noise being converted to position noise anyway. The cantilever amplitude signal is measured as described above. No filtering is applied to the amplitude signal.

The post-processing in this work is extended as follows. From the piezo position signal, representing the tip–sample distance, a $\kappa_{\mathrm{ts}}$-vector is calculated (details follow, see (12) and (15)). In addition, the amplitude responsivity is calculated using the gradient function in Matlab. Because the amplitude signal contains noise, the gradient calculation yields unwanted results. Therefore, the amplitude signal is smoothed using a quadratic regression (smoothdata with loess) and smoothing spline algorithm (fit with smoothingspline) from Matlab.

## 3. Results

### 3.1. Comparison of Parametric Excitation Schemes

**Figure 2:**
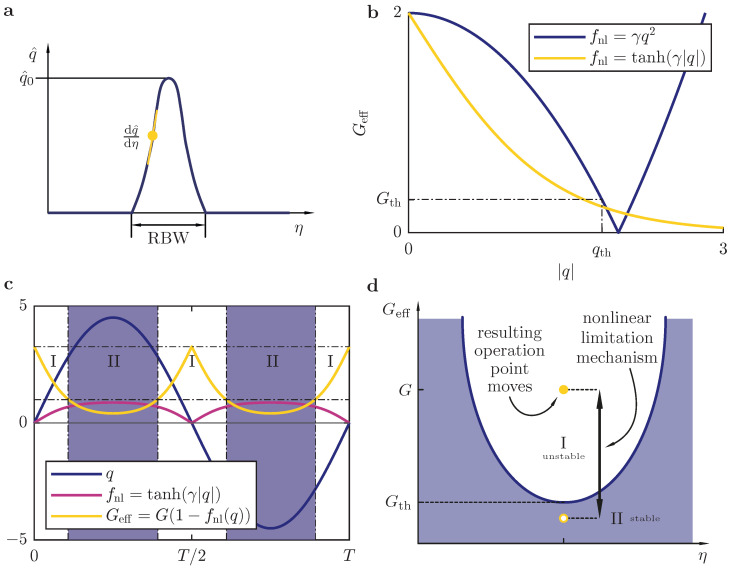
Characteristics of amplitude response curves and illustration of nonlinear limitation mechanism. (**a**) Exemplary amplitude response curve of a parametric oscillator with characteristics free amplitude ${\hat{q}}_{0}$, resonance bandwidth (RBW), and slope $d\hat{q}/d\eta$. (**b**) Parametric excitation amplitude $G_{\mathrm{eff}}=G\left(1-f_{\mathrm{nl}}\left(q\right)\right)$ as contained in (10) as absolute value of oscillator displacement |q| increases. Blue curve: $f_{\mathrm{nl}}=\gamma x^{2}$. For |q|>2, the parametric excitation amplitude increases and can cause instability. Yellow curve: $f_{\mathrm{nl}}=\tanh\left(\gamma |q|\right)$. Converges to $G_{\mathrm{eff}}=0$ for large |q|. (**c**) ODE simulation of exemplary cantilever oscillation *q* (blue) over one oscillation period *T* with G=3.26, γ=0.3, η=2. Corresponding nonlinear limitation function $f_{\mathrm{nl}}$ (pink) and effective parametric excitation amplitude $G_{\mathrm{eff}}$ (yellow). Regions I mark excitation above threshold for parametric resonance, $G_{\mathrm{eff}}>G_{\mathrm{th}}$. Regions II mark $G_{\mathrm{eff}}<G_{\mathrm{th}}$. (**d**) Corresponding instability chart with resulting operation point moving up and down, outside (II) and inside (I) of instability tongue.

In this section, different excitation schemes are compared. The goal is to find a suitable equation structure that enables steep resonance curves in order to enhance the sensitivity and, in general, a flexibility, meaning that different characteristics can be adjusted independently. First, these characteristics are defined. For that, Figure 2a shows an exemplary amplitude response curve of a parametric oscillator. We define three main characteristics of an amplitude response curve. The first is the *free amplitude* ${\hat{q}}_{0}$, which is the amplitude in parametric resonance with negligible tip–sample interaction, also see [4]. The second characteristic is the *resonance bandwidth (RBW)* that marks the width of the parametric resonance at $\hat{q}=0$. The last and most important characteristic is the *slope of the amplitude response curve $d\hat{q}/d\eta$*, which should be as high as possible and which contributes to high amplitude responsivity [4]. Additionally, a more qualitative characteristic is the position along the amplitude response curve where the slope is the steepest. For all characteristics, there are no specific values to reach. Nevertheless, it is important to define them in order to systematically compare and evaluate different excitation schemes.

As indicated in Figure 2a, we aim to get symmetric amplitude response curves to have a more beneficial system behavior. Asymmetric curves may have unstable branches on which the system cannot be operated. We want to choose the operating point on the curve freely without any limitation because of instability. Furthermore, the corresponding stable branch will always be less steep compared to the unstable branch. So, we already see an improvement of the slope and with that of the amplitude responsivity by having symmetric amplitude response curves. Because of that, we sort out systems that lead to the nonlinear Mathieu Equation (4) and the Mathieu–Duffing Equation (5).

Now, we take a deeper look at the system investigated by Prakash et al. where symmetric curves are achieved [12] (Figure 2). The difference of that system, [12], compared to other systems [14,15,16,17,18,19,20,21,22] is that the origin of the nonlinearity in [12] is in the displacement detection system of the cantilever. So, this nonlinearity does not directly limit the amplitude of the system; rather, it determines the saturation of the parametric excitation of the cantilever and with that causes the limitation of the amplitude. So, the cantilever itself in [12] is a linear system with an amplitude that would grow unboundedly in parametric resonance, but which is limited due to a saturation of the parametric pumping. This concept is an entirely different amplitude limitation mechanism compared to [14,15,16,17,18,19,20,21,22]. Here, this mechanism is first analyzed and explained and then exploited in this work.

In contrast to the analyzed literature, we are not limited to a cubic nonlinearity, as we design the system to include the nonlinear behavior on purpose. Therefore, we can search for other types of nonlinearity that show better behavior than the previously used cubic type. In order to adopt a systematic approach, we state a generalized system equation by introducing an arbitrary nonlinear function $f_{\mathrm{nl}}\left(q\right)$ that limits the parametric excitation amplitude of the parametric oscillator,

(10) $$q^{\prime \prime }+2Dq^{\prime }+q\left[1+G\left(1-f_{\mathrm{nl}}\left(q\right)\right)\cos\eta \tau \right]=0,$$

where $G_{\mathrm{eff}}=G\left(1-f_{\mathrm{nl}}\left(q\right)\right)$ is the effective parametric excitation amplitude and *G* the excitation amplitude set as operation point. There is parametric excitation with a resulting oscillation in parametric resonance if $G_{\mathrm{eff}}>G_{\mathrm{th}}$. This condition is satisfied for increasing *q* as long as $f_{\mathrm{nl}}\left(q\right)<1-G_{\mathrm{th}}/G$. Otherwise, if *q* increases further, there is $f_{\mathrm{nl}}\left(q\right)\geq 1-G_{\mathrm{th}}/G$ and with that $G_{\mathrm{eff}}\leq G_{\mathrm{th}}$. Figure 2c,d illustrate this nonlinear limitation mechanism. In c, an exemplary harmonic function representing the cantilever oscillation is shown (blue curve) over one oscillation period *T*. The pink curve is such a nonlinear limitation function, here $f_{\mathrm{nl}}\left(q\right)=\tanh\left(\gamma |q|\right)$ as an example. The yellow curve shows the effective parametric excitation amplitude along one oscillation period. There exist two types of region, I and II. In I, the parametric excitation amplitude is smaller than the threshold for parametric resonance, $G_{\mathrm{eff}}\leq G_{\mathrm{th}}$. II is the opposite, $G_{\mathrm{eff}}>G_{\mathrm{th}}$. Because the system switches between both states several times during one oscillation period, there will be a steady state resulting in a stable oscillation. This is also illustrated in the instability chart, Figure 2d. Here, we see this switching as the resulting operation point moves up and down, respectively, outside (region II) and inside (region I) of the parametric instability tongue.

To determine $f_{\mathrm{nl}}$, we set $f_{\mathrm{nl}}=\gamma q^{2}$ as a first step. This gives the same system equation developed before [12] (Equation (2)) with the additional option to adjust the limitation strength by γ. For better understanding, Figure 2b shows the parametric excitation amplitude $G_{\mathrm{eff}}$ over the absolute value of the oscillator displacement |q| with the blue curve representing $f_{\mathrm{nl}}=\gamma q^{2}$. The resulting displacement of the parametric oscillator where the parametric excitation amplitude is $G_{\mathrm{th}}$ is $q_{\mathrm{th}}=\left(1-G_{\mathrm{th}}/G\right)/\gamma$. Here, a problem arises as $q>q_{\mathrm{th}}$, i.e., by a perturbation of any kind during operation or due to the oscillatory behavior of the system. As a consequence, the absolute value of the excitation amplitude could increase again, $|G_{\mathrm{eff}}|>G_{\mathrm{th}}$, which, in fact, just leads to further parametric pumping with $G_{\mathrm{eff}}>G_{\mathrm{th}}$ and results in instability. This is possible because the system itself does not have nonlinear stiffness. To avoid this, we need to find a nonlinear function $f_{\mathrm{nl}}$ that leads to a convergence of the excitation amplitude for large cantilever amplitudes, $\lim_{q\to \infty }G_{\mathrm{eff}}=0$.

Such a function is, for example, $f_{\mathrm{nl}}=\tanh\left(\gamma |q|\right)$ (yellow curve in Figure 2b). With that, instability caused by perturbations is not possible. On this basis, we choose the hyperbolic tangent function as the nonlinear limitation function. Doing this, we finally obtain

(11) $$q^{\prime \prime }+2Dq^{\prime }+q\left[1+G\left(1-\tanh\left(\gamma |q|\right)\right)\cos\eta \tau \right]=0$$

as a system equation with the excitation amplitude *G* and the nonlinearity factor γ. In the next section, this system is analyzed in order to get an overview about the different parameters influencing the system behavior.

### 3.2. Numerical Analysis

#### 3.2.1. General System Analysis

**Figure 3:**
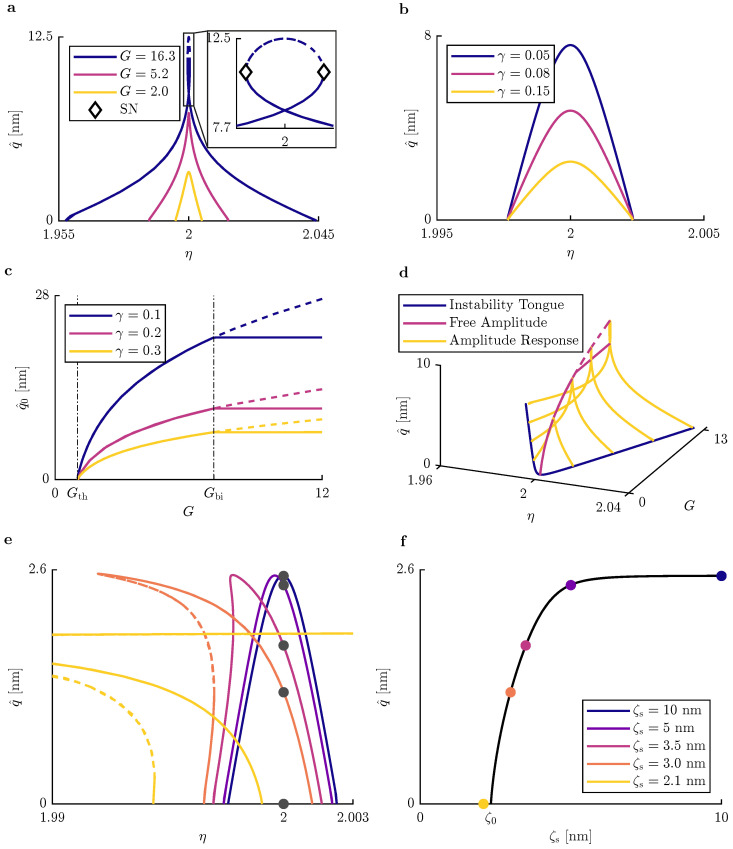
Numerical system analysis including approach mechanism. Amplitude response curves for varying excitation parameters, (**a**): variation of excitation amplitude *G* while γ=0.25 including bistable behavior for large *G*. White diamonds mark saddle nodes. (**b**) Variation of nonlinearity factor γ while G=1.31. (**c**) Free Amplitude ${\hat{q}}_{0}$ in dependence of excitation amplitude *G* for different nonlinearities γ. Two thresholds $G_{\mathrm{th}}$, $G_{\mathrm{bi}}$ mark the beginning of parametric resonance and the beginning of bistable behavior. (**d**) Overview of system dynamics showing amplitude response curves, free amplitude with bistable behavior and the instability tongue (γ=0.3). (**e**) Amplitude response curves for varying quasistatic tip–sample distance $\zeta_{s}$. For small distances, the nonlinear behavior of the tip–sample interaction force $F_{\mathrm{ts}}$ gets visible through the bending of the amplitude response curves. (**f**) Corresponding amplitude–distance curve. Both together show the approach mechanism of the parametrically excited AFM system. Parameters are ${\hat{q}}_{0}=10$ nm, G=1.31, γ=0.15, (η=2).

In order to analyze the selected excitation scheme (11), the influence of the parameters *G* and γ is investigated. Therefore, amplitude response curves for several parameter values are calculated using numerical continuation (for further details see Section 2). These are shown in Figure 3a,b. Note, that the trivial solution which is stable outside and unstable inside the parametric resonance is not calculated explicitly here. Figure 3a shows that for increasing excitation amplitude *G*, the resonance bandwidth increases, as well as the free amplitude ${\hat{q}}_{0}$. Additionally, the general shape of the curve changes. The gradient along the arclength of the curve is less constant meaning that the steepness at the begin of the parametric resonance is lower compared to smaller values of *G*. Going further towards η=2, the steepness increases. In the vicinity of η=2, bistable behavior appears for large values (G=16.3). Additionally, a third, unstable solution branch exists, marked by the saddle-nodes (SN).

In contrast, Figure 3b shows the influence of the nonlinearity factor γ. Here, it can be seen that the shape of the curves and the resonance bandwidth is not influenced by γ but only the free amplitude. So, with the nonlinearity factor, there is a simple way of adjusting the resulting free amplitude without modifying the rest of the curve. Nevertheless, it is already clear that with this method, the steepness also changes.

To gain better insight, Figure 3c shows the dependence of the free amplitude ${\hat{q}}_{0}$ on the excitation amplitude *G* for different nonlinearity factors γ. Two characteristic excitation amplitudes can be observed, named $G_{\mathrm{th}}=1$ and $G_{\mathrm{bi}}=7.13$. The first marks the threshold for parametric resonance, meaning that for lower excitation amplitudes no stationary oscillation occurs. Instead, oscillations of the system decay to the trivial solution. This is common knowledge and, i.e., stated in [10]. The second threshold is of more interest as it marks the begin of bistable behavior (see the loop in the amplitude response for G=16.3 in Figure 3c). This was previously observed in a similar system [24]. From $G_{\mathrm{bi}}$ on, the free amplitude stays at a constant value. Even if we do not see any practical advantage to bistability in our case, this is an important regime as there is a risk of jumping between the solution branches, which needs to be prevented in the later operation of the system. So, the excitation amplitude needs to be $G_{\mathrm{th}}<G<G_{\mathrm{bi}}$ in order to stay in a safe operation regime.

We see that it is not possible to adjust all the desired characteristics completely independently of each other. So, all parameters should be considered at once. For this, Figure 3d shows an overview of the system dynamics. For parameters η, *G*, and γ amplitude response curves, the free amplitude curve and the edge of the instability tongue is shown for a value γ=0.3. It can be seen how the amplitude response curves are connected by the free amplitude curve. The beginning of the parametric resonance is marked by the edge of the instability tongue.

#### 3.2.2. Approach Mechanism

To continue the dynamic analysis, we include the tip–sample force to gain an understanding of the amplitude reduction mechanism during the approach. The interaction force model was previously used [4] (Equation (2)) and follows [37,38], (Equation (1))

(12) $$F_{\mathrm{ts}}\left(\zeta \right)=\frac{AR}{6}\left(\frac{1}{\zeta^{2}}-\frac{a_{0}^{6}}{30}\;\frac{1}{\zeta^{8}}\right),$$

with the Hamaker constant *A*, the tip radius of the cantilever *R*, the intermolecular distance of the sample molecules $a_{0}$, and the tip–sample distance ζ. The dominant force included in this model is the van der Waals force [39]. Inserting (12) in (11) leads to the equation of motion of the cantilever under the influence of the tip–sample force,

(13) $$q^{\prime \prime }+2Dq^{\prime }+q\left[1+G\left(1-\tanh\left(\gamma |q|\right)\right)\cos\eta \tau \right]=F_{\mathrm{ts}}\left(\zeta \right).$$

Now, we calculate several amplitude response curves at different quasistatic tip–sample distances $\zeta_{s}$ (details in Section 2). Similarly, an amplitude distance curve is computed as well. Both are shown in Figure 3e,f. We must keep in mind that the excitation frequency is fixed at η=2. Approaching the sample, it can be seen that the amplitude response curves are first shifted to the left. This is the same result as operating the AFM with forced excitation and can be explained by the assumption of a linearized tip–sample force, the so-called tip–sample stiffness [3], which we used before to analyze the forced excited AFM [4]. Approaching further, we see that this assumption no longer holds as the amplitude response curves start to bend to towards lower frequencies. Here, the nonlinear character of the tip–sample force gets visible. In any case, the gray points indicate the amplitude values at excitation frequency η=2. These points can be found in the corresponding amplitude–distance curve, where they are colored corresponding to the amplitude response curves. If the cantilever is further approached to the sample, we observe that at a specific distance the amplitude is at zero but with still a significant tip–sample distance left (here $\zeta_{0}\approx 2.35$ nm). This can be explained with the plot in Figure 3e, where we see that the amplitude response curve for $\zeta_{s}=2.1$ nm shifted to the left, so the excitation frequency lies outside the parametric resonance. This phenomenon is a significant difference to the forced excited AFM, where the amplitude is always zero when the tip–sample distance is zero as well. We see here an advantage regarding the general operation of the AFM system because that phenomenon serves as indication that it should be impossible to touch the sample with the tip during operation which prevents tip and/or sample damage (under the assumption that the approach ends when the amplitude is zero and that the proposed tip–sample force model, Equation (12), is the dominant interaction force). Of course, this still needs to be further proved experimentally. If $\zeta_{0}$ needs to be adjusted for some reason, this can be done by adjusting the resonance bandwidth with parameter *G* as shown above.

#### 3.2.3. Parameter Influence on Amplitude Noise

**Figure 4:**
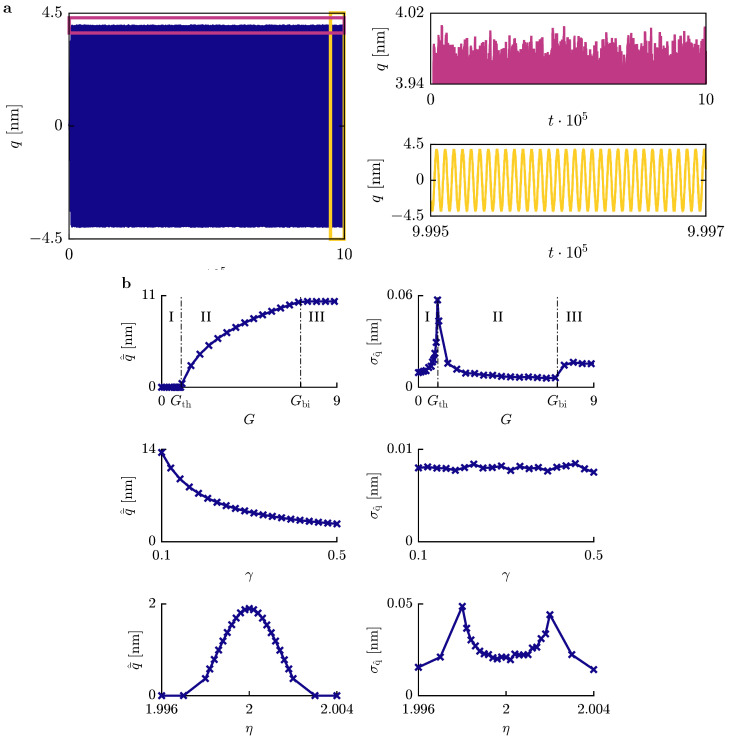
Parameter influence on amplitude noise. (**a**) Stationary time series of system in parametric resonance. Oscillations seems to be purely harmonic. Zoom in shows the amplitude fluctuations due to thermal noise force. Parameters: G=1.97, γ=0.21, η=2. (**b**) Mean and standard deviation of cantilever amplitude, influenced by excitation parameters. First row: variation of excitation amplitude *G* while γ=0.21 and η=2. Regions I mark parametric amplification and regions II and III mark parametric resonance. Second row: variation of nonlinearity factor γ while G=3.26 and η=2. Third row: variation of excitation frequency η while G=1.31 and γ=0.2.

As next part of the system analysis, the behavior of amplitude noise of the parametrically excited AFM system should be investigated under the influence of the excitation parameter *G*, γ and η. Before going into the analysis, we first have to determine if the condition of small noise is satisfied to assume a Gaussian process and use the statistical properties of variance and mean for analysis, as explained earlier. For that, the system (11) is expanded with the thermal noise force, resulting in

(14) $$q^{\prime \prime }+2Dq^{\prime }+q\left[1+G\left(1-\tanh\left(\gamma |q|\right)\right)\cos\eta \tau \right]=F_{\mathrm{th}},$$

where the tip–sample interaction force is neglected as it does not influence the noise behavior. Other potential noise sources originating in the experimental realization of (14) that would appear as multiplicative noise are neglected as we only investigate thermal noise as a physically caused limitation of sensitivity and no electronic noise. The thermal noise is, instead, purely additive. Then, Equation (14) is numerically integrated. A detailed description of the numerical procedures can be found in Materials and Methods. Figure 4a shows an exemplary stationary time series of the system in parametric resonance. It can be seen that the deterministic behavior of the system is dominating, meaning that the response seems to be purely harmonic as expected (yellow zoom) [10]. Just if we zoom in and have a closer look, we see that the amplitude of the oscillations fluctuates (pink zoom). Because the fluctuations are small compared to the mean value of the amplitude (in this example, a ratio $\sigma_{\hat{q}}/\hat{\bar{q}}\leq 3.5\cdot 10^{-3}$ with standard deviation of the oscillator amplitude $\sigma_{\hat{q}}$), we see the condition of small noise as satisfied and continue the analysis assuming the system response to be Gaussian.

To start, we consider the influence of the excitation parameters on the amplitude fluctuations of the system under the impact of the thermomechanical noise, as shown in Equation (14). For varying excitation parameters time series as in Figure 4a are calculated, which are statistically evaluated to get the RMS values of the amplitude noise $\delta \hat{q}$. The results are shown in Figure 4b. In the left column, the mean values of the tip displacement amplitude $\hat{\bar{q}}$ are plotted for varying parameters *G*, γ, η. In the right column, the same is done for the standard deviation of the amplitude $\sigma_{\hat{q}}$ values, which represent the RMS value of the amplitude noise, $\sigma_{\hat{q}}=\delta \hat{q}$. The plots of the mean value are identical to the previously computed plots; see Figure 3a–d. This shows that the influence of the thermomechanical noise on the system behavior is small and that the macroscopic behavior is the same as that of the deterministic system without noise. For example, the mean values for a variation of the excitation amplitude *G* are identical to Figure 3c where the free amplitude is plotted (only stable branches). Also, the means for a variation of the excitation frequency η are identical to the values in Figure 3a,b.

More interesting is the standard deviation $\sigma_{\hat{q}}$ (RMS value of $\delta \hat{q}$) for different parameters. Looking at the first row, it can be seen that $\sigma_{\hat{q}}$ increases for increasing $G<G_{\mathrm{th}}$ (region I). Reaching the threshold for parametric resonance, $G_{\mathrm{th}}$, the standard deviation diverges to infinity. In parametric resonance (region II), $G>G_{\mathrm{th}}$, $\sigma_{\hat{q}}$ decreases and seems to converge to $\sigma_{\hat{q}}=0$. Until now, this behavior was previously found by Li [32] (Equation (3.61)). At the second threshold $G_{\mathrm{bi}}$ where the bistable behavior occurs, the standard deviation jumps to a fixed value, $\sigma_{\hat{q}}\approx 0.015$ (region III). So, in order to minimize the resulting standard deviation of the amplitude, the excitation amplitude *G* should be close to the second threshold $G_{\mathrm{bi}}$ but not above. Anyway, it should be noticed that near $G_{\mathrm{th}}$ the small-noise assumption technically does not hold, and therefore a Gaussian distribution of the amplitude noise cannot be directly ensured. Nevertheless, as already explained, previous studies have made use of variance analysis, particularly at the threshold of parametric resonance. Since this paper focuses primarily on the operation of the system within the parametric resonance and not at its edge, we will not discuss the validity of a Gaussian distribution at the threshold in further detail and refer to the state of the art.

For a variation of the nonlinearity factor γ, it can be seen that the amplitude noise is not influenced.

The behavior for a variation of the excitation frequency η shows minimum noise for η=2. Below and above that value, the behavior is symmetric to η=2, meaning an increase of $\sigma_{\hat{q}}$ towards the edges of parametric resonance where $\sigma_{\hat{q}}$ diverges. This is not surprising considering the first row of Figure 4b, where we can also observe divergent behavior at the edge of parametric instability.

So, to conclude, only *G* and η influence the resulting amplitude fluctuations due to thermal noise. This leads to the insight that the operation point in the instability chart determines the amplitude noise, not the deterministic amplitude, which is influenced by γ.

#### 3.2.4. Parameter Influence on Amplitude–Distance Curves

In order to deeply analyze the system behavior including the tip–sample interaction force, the complexity of the model is reduced. Therefore, the interaction of the cantilever tip and the sample, consisting of the tip parameters and the sample parameters, is reduced to a single parameter $\kappa_{\mathrm{ts}}$ as done in [4] (Equation (17)). In detail, the tip–sample interaction force (12) at a given quasistatic tip–sample distance is linearized to obtain the tip–sample stiffness $k_{\mathrm{ts}}\left(\zeta \right)$. This assumes a quasistatic tip–sample distance $\zeta_{s}$, with the effect of the actual oscillation—mainly, the variation of the tip–sample distance around $\zeta_{s}$—being negligible. We consider this justified by the nature of amplitude–distance curves under parametric excitation, for which ${\hat{q}}_{0}=0$ is reached well before $\zeta_{s}=0$. Due to this, the cantilever amplitude is zero before the tip–sample interaction force becomes strongly nonlinear at smaller tip–sample distances; see Figure 3e,f. In other words, parametrically excited AFM supports the operation in non-contact mode AFM. If that holds for all parameter combinations must me part of future investigations. It can be assumed that limitations are regarding wider amplitude response curves but quite independent of the value of the cantilever amplitude. In contrast, the approximation of $F_{\mathrm{ts}}$ with $\kappa_{\mathrm{ts}}$ for forced excited AFM is certainly only possible for small amplitudes [4,40].

The tip–sample stiffness is normalized to the stiffness *k* of the cantilever, which yields the dimensionless tip–sample stiffness

(15) $$\kappa_{\mathrm{ts}}\left(\zeta_{s}\right)=\frac{k_{\mathrm{ts}}\left(\zeta_{s}\right)}{k}.$$

This quantity is an abstraction of the strength of the tip–sample interaction and allows us to analyze the influence of the excitation parameters on the system behavior without limitation to a specific cantilever and/or sample. By this, the results gained in this contribution apply to any combination of cantilevers and samples and thus represent a generalized overview of the behavior of parametric resonance AFM [4].

**Figure 5:**
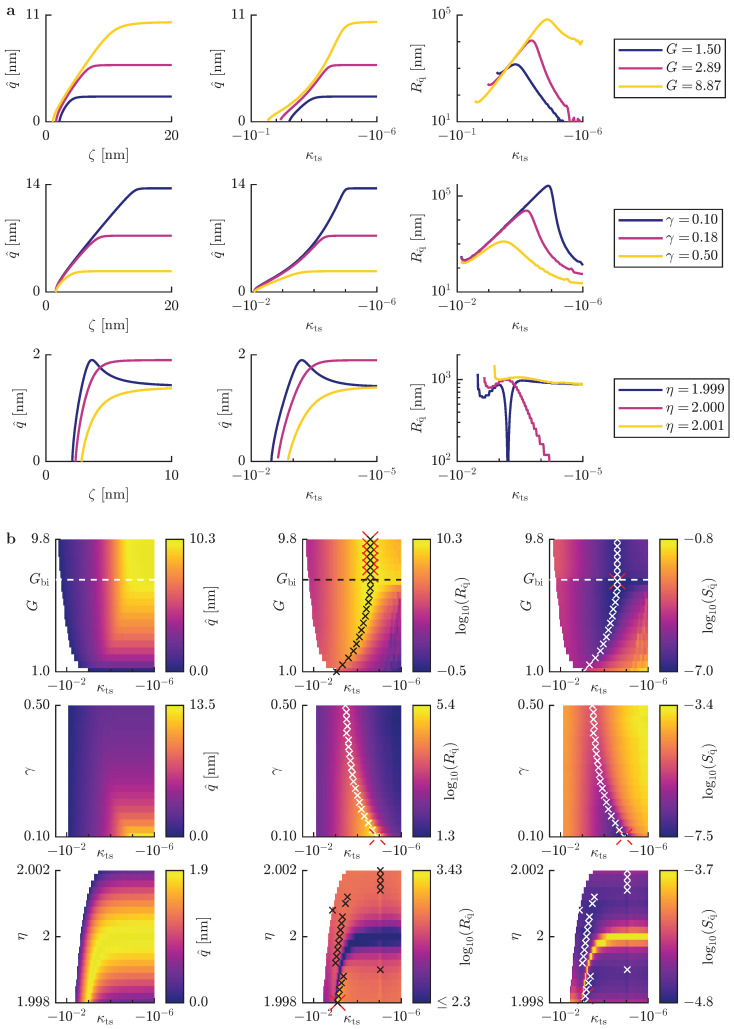
System behavior influenced by tip–sample interaction and noise. (**a**) Amplitude–distance curves, amplitude over dimensionless tip–sample stiffness $\kappa_{\mathrm{ts}}$, and amplitude responsivity $R_{\hat{q}}$ for varying excitation parameters. In the first row, variation of excitation amplitude *G*, while γ=0.21 and η=2. In the second row, variation of nonlinearity factor γ, while G=3.26 and η=2. In the third row, variation of excitation frequency η, while G=1.31 and γ=0.2. (**b**) Overview of amplitude $\hat{q}$, amplitude responsivity $R_{\hat{q}}$, and amplitude sensitivity $S_{\hat{q}}$ influenced by the parametric excitation parameters. White and black crosses mark local maxima of $R_{\hat{q}}$ and minima of $S_{\hat{q}}$. Red crosses mark global maxima/minima per plot. First row: variation of excitation amplitude *G*, while γ=0.21 and η=2. Second row: variation of nonlinearity factor γ, while G=3.26 and η=2. Third row: variation of excitation frequency η, while G=1.31 and γ=0.2.

To start the analysis of how the excitation parameters *G*, γ, and η influence the AFM system, we display several approach curves with varying excitation parameters. Figure 5a shows three columns. In the first column, amplitude–distance curves can be seen. The second column contains the amplitude over the dimensionless tip–sample stiffness $\kappa_{\mathrm{ts}}$. In the third column, the amplitude responsivity $R_{\hat{q}}$ is shown. Looking to the first row, we see how the curves develop for increasing values of the excitation amplitude *G*. We can clearly observe the effects shown in Figure 3a: increasing *G* leads to wider and higher amplitude response curves, which then affect the amplitude–distance curves in such a way that the characteristic quasistatic tip–sample distance $\zeta_{0}$ where the amplitude is zero decreases for increasing *G*. Moving to the second column, it can be seen that the general shape of the curves changes for increasing *G*. For lower values of *G*, the shape is the same as for the amplitude–distance curves, meaning convex, while for higher values of *G*, a concave shape appears. More interestingly is the fact that the tip–sample distance where the amplitude reduction starts shifts towards smaller values of $\kappa_{\mathrm{ts}}$ for increasing *G*. This leads to higher amplitude responsivities $R_{\hat{q}}$ as shown in the third column. This makes sense because, here, obviously less tip–sample interaction is necessary to cause a change or reduction of the amplitude. This fact is also interesting regarding the operational stability of the AFM system, since in general larger cantilever amplitudes are better to control.

For a variation of the nonlinearity factor γ, we see that $\zeta_{0}$ is not influenced by γ, remaining at a constant value. Remembering Figure 3b, this makes sense, as γ does not change the resonance bandwidth of the amplitude response. If we look at columns two and three, we see that the general behavior is similar to the curves for a variation of *G*.

In the last row, the excitation frequency η is varied. Remarkably, the resulting behavior is comparable to that of an AFM system with forced excitation, which we analyzed earlier [4] (Figure 3b): for an excitation frequency η<2, exciting below resonance, the amplitude first increases and then decreases while approaching the sample. This was explained previously with the shift of the resonance frequency during approach, and is well known. In contrast, for an excitation frequency η>2, the free amplitude is already lower before the approach. Looking at the amplitude responsivity, we see that the highest value (absolute value) appears for an excitation frequency η>2, which marks a difference to a forced excited system, where the largest amplitude responsivity is gained for η<2.

Now, to get a global overview of the influence of parameters *G*, γ, and η, we move to a three-dimensional representation of the system behavior and additionally include the previously calculated results of the influence of the parameters on the amplitude noise of the cantilever. This leads to an understanding of the influence of the parameters *G*, γ, and η on the amplitude sensitivity $S_{\hat{q}}$ (resolution) of parametrically excited AFM. The analysis is done for each of the parameters *G*, γ, and η separately and consists of three-dimensional plots, as shown in Figure 5b, showing the amplitude $\hat{q}$, amplitude responsivity $R_{\hat{q}}$, and amplitude sensitivity $S_{\hat{q}}$ over a varied parameter and increasing dimensionless tip–sample stiffness $\kappa_{\mathrm{ts}}$. This kind of plot was introduced before [4] (Figure 4) and is now adapted to the current system.

Before going into a deeper analysis, we want to clarify the connection between Figure 5a,b. Each line in Figure 5a is part of Figure 5b, represented as a horizontal line with constant *G*, γ, and η and the function value color-coded into the third dimension (see [4] for comparison). By increasing the number of single lines, we arrive at this three-dimensional representation.

Starting with a variation of *G* and looking at the amplitude plot, we find the previously observed behavior, including the change of $\zeta_{0}$ with *G* and the bistable regime, whose beginning is marked by the dashed line. Looking at the amplitude responsivity plot, a local maximum can be seen along $\kappa_{\mathrm{ts}}$ for each value of *G*, marked by the black crosses.

For increasing *G* starting from $G_{\mathrm{bi}}$, the maximum values of the amplitude responsivity converge as well. So, several red crosses mark this global maximum line. From this point of view, values of $G\geq G_{\mathrm{bi}}$ give the highest amplitude sensitivity. Moving to the amplitude sensitivity by including the noise data shown in Figure 4b according to (2) shows that the general shape seems to be identical to the shape of the amplitude responsivity. The important difference is that the sensitivity has a global minimum and not a minimum line as the amplitude responsivity, marked by only one red cross. The minimum and therefore best value is directly before $G_{\mathrm{bi}}$. This corresponds to the noise behavior in Figure 4b.

The variation of γ is shown in the second row of Figure 5b. We again see that $\zeta_{0}$ is not influenced by γ since all the single amplitude–distance curves in this plot end at the same value $\kappa_{\mathrm{ts}}\approx 8\cdot 10^{-3}$. In the second column, it can be seen that the amplitude responsivity increases with decreasing γ. In any case, the highest responsivity for each γ is directly after the beginning of the amplitude reduction with increasing $\kappa_{\mathrm{ts}}$. So, small γ gives high responsivity. Because the noise level is constant over γ, the amplitude sensitivity behaves the same, meaning that small γ yields the best sensitivity.

When the excitation frequency η is varied, the general behavior is identical to a forced excited system [4]. There are dedicated maximums in the amplitude sensitivity for each η and the amplitude responsivity has two maximum lines, above and below the resonance frequency (ignoring the numerical artifacts at $\kappa_{\mathrm{ts}}=-10^{-5}$). In principle, the amplitude sensitivity behaves the same regarding two minimum lines. However, there are also significant differences compared to a forced excited system. First, the overall variance of amplitude responsivity in the investigated parameter field is not high. That can be seen by the fact that most of the plot is constantly orange. As expected from the forced excited system, there is no region with low responsivity for frequencies more far away from the resonance (η≤1.997 and η≥2.003), because in the case of the parametrically excited system, there is no parametric resonance anymore. Second, maximum responsivity is gained for η=1.998. This originates in the noise behavior. The high responsivity below the resonance frequency has a high noise level as a counterpart; see Figure 4b. So, taking into account the amplitude responsivity and the noise, we have best sensitivity in the vicinity of the resonance frequency.

To summarize the numerical study, we calculate for the parameters *G*, γ, and η the values of $\kappa_{\mathrm{ts}}$ where the amplitude sensitivity is best. With that information, it is possible to optimize an existing AFM system regarding cantilever and sample parameters to gain the desired value of $\kappa_{\mathrm{ts}}$ to get best sensitivity when applying the proposed parametric excitation mode.

### 3.3. Experimental Validation

To conclude our analysis, experiments are carried out in order to validate the numerical findings. For this, we measure the amplitude response curves of a parametrically excited cantilever for a variation of the excitation parameters presented. Furthermore, amplitude–distance curves are measured using a setup we presented before [4]. It should be already noted that the cantilever used in the experiments has a resonance frequency of approximately 62 kHz. The excitation parameters, namely *G* and γ, are set by the control registers of an FPGA and are referred to as C1 and C3 in this experimental section. The exact measurement setup, including the feedback loop for generating the nonlinear parametric excitation and the postprocessing procedure of the measured data, is described in Materials and Methods. In addition, we want to clarify that the experiments carried out in this work have the goal of validating our numerical findings qualitatively. In order to reproduce the exact numerical values, we need to fully characterize the experimental setup including all parts of the FPGA and amplifiers used. This should be postponed to future work.

**Figure 6:**
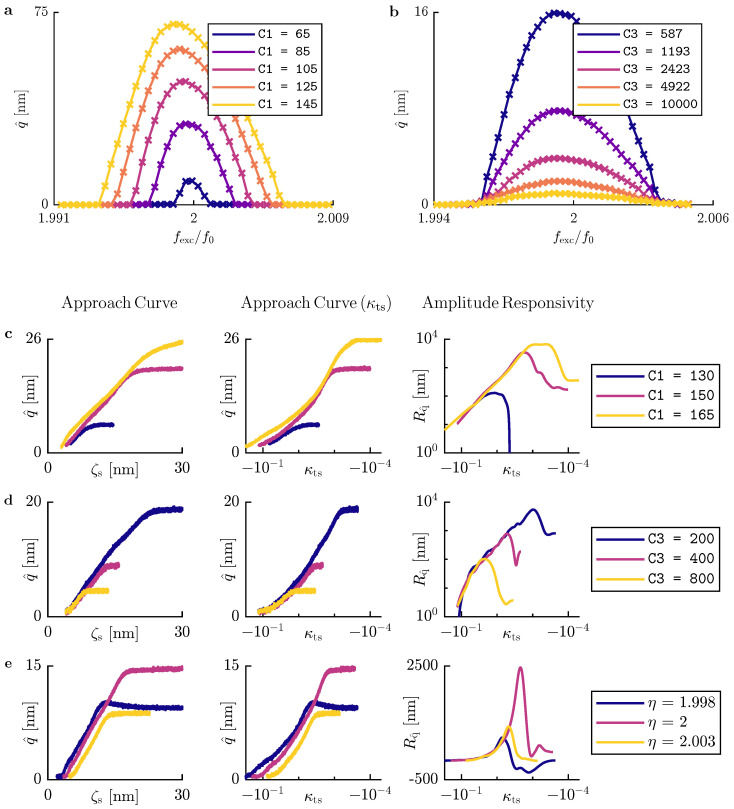
Experimental results of deterministic system. (**a**,**b**) Experimental amplitude response curves for varying excitation parameters show identical qualitative behavior as numerical findings, Figure 5. (**a**) Excitation amplitude *G*, represented by control register C1. C3 = 200. (**b**) Nonlinearity factor γ, represented by control register C3. C1 = 100. (**c**–**e**) Experimental amplitude–distance curves, amplitude over dimensionless tip–sample stiffness $\kappa_{\mathrm{ts}}$ and amplitude responsivity $R_{\hat{q}}$ for varying excitation parameters show identical qualitative behavior as numerical findings, Figure 5a. (**c**) Variation of excitation amplitude *G*, represented by control register C1, while C3 = 200 and η=2. (**d**) Variation of nonlinearity factor γ, represented by control register C3, while C1 = 190 and η=2. (**e**) Variation of excitation frequency η, while C1 = 190 and C3 = 300.

Figure 6a shows experimental amplitude response curves for a variation of C1 (*G*), and Figure 6b shows the same for a variation of C3 (γ). Comparing these with the numerical counterparts in Figure 3a,b, we see that we observe the exact same behavior of how the excitation parameters influence the characteristics of the amplitude response curves.

Moving to Figure 6c–e, where experimental amplitude–distance curves for varying parameters *G*, γ, and η are shown. Varying the excitation amplitude *G* in Figure 6c, the same behavior as in the simulation is present, meaning the change of the curve shape from convex to concave, as well as the increasing amplitude responsivity with increasing *G*. The same holds for the variation of the nonlinearity factor γ in Figure 6d, where the amplitude responsivity increases for decreasing γ. Figure 6e shows a variation of the excitation frequency η. Looking to the first and second plot, we see the same qualitative behavior as presented in Figure 5a. For η<2, the amplitude increases first during approach. Nevertheless, we see that this effect is not as strong as predicted from the simulation. The reason for this was previously found [4] (Figure 2). In contrast to the simulation, where steady-state responses are calculated, in the experiment the cantilever approaches the sample with a velocity $v_{A}\neq 0$. Due to this, there is not enough time for the transients to die out and the cantilever amplitude to reach the steady-state value. Therefore, the effect is less, meaning that the increase of the amplitude is not as much as simulated. Looking to the amplitude responsivity, the qualitative behavior as shown in Figure 5a can also be found again. The difference is that the blue curve (that one for η<2) is not as steep as expected, as we can see in the reduced amplitude responsivity. This, of course, also originates from $v_{A}\neq 0$.

**Figure 7:**
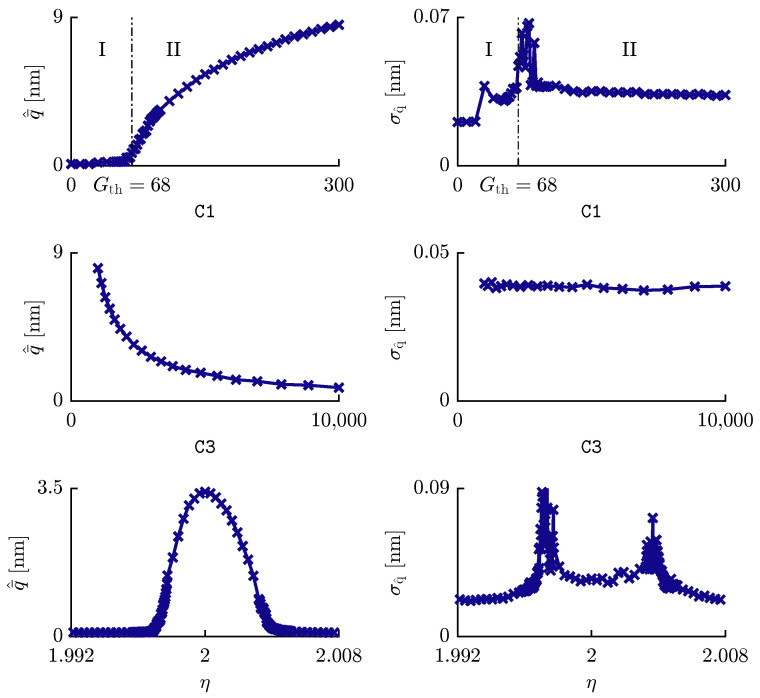
Experimentally determined mean and standard deviation of cantilever amplitude noise, influenced by excitation parameters showing the same qualitative behavior, is in Figure 4b. First row: variation of excitation amplitude *G*, represented by control register C1, while C3 = 2423 and η=2. Regions I mark parametric amplification and regions II mark parametric resonance. Second row: variation of nonlinearity factor γ, represented by control register C3, while C1 = 100 and η=2. Third row: variation of excitation frequency η, while C1 = 100 and C1 = 2423.

Lastly, the simulation of the amplitude noise, shown in Figure 7, should be experimentally validated. Therefore, several parameter sweeps are statistically evaluated (see Section 2). The results in Figure 7 show agreement with the previous simulations in Figure 4b and reveal again the significant increase in the standard deviation of the cantilever amplitude at the edge of parametric resonance. It should be noted that for the variation of the excitation amplitude C1 (*G*), the measured parameter range corresponds to small values of *G* in Figure 4b and does not show features for larger *G* as the bistable behavior, which is supposed to be missing region III. This is due to actuator limitations in the experiment, which do not allow stronger excitation to get to the bistable behavior.

To conclude, all numerical simulations are validated by experiments, and now a comparison of the conventional, forced excited AFM system with the parametrically excited AFM presented here can be performed.

### 3.4. Comparison to Conventional Forced Excitation

**Figure 8:**
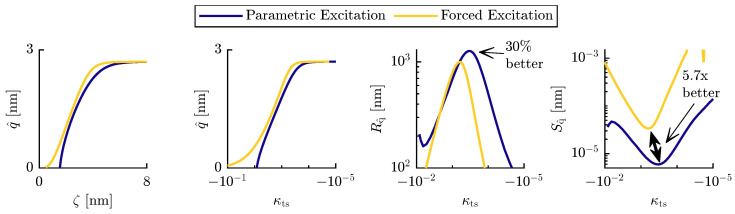
Comparision of parametric resonance AFM and conventional AFM with forced excitation showing amplitude–distance curves, amplitude over dimensionless tip–sample stiffness $\kappa_{\mathrm{ts}}$, amplitude responsivity $R_{\hat{q}}$ and amplitude sensitivity $S_{\hat{q}}$ for both excitation modes. Parameters for parametric resonance AFM: G=3.26, γ=0.5, η=2. An improvement of amplitude responsivity of 30% for this specific parameter set can be found using parametric resonance AFM, while the amplitude sensitivity is increased even by a factor 5.7.

For a comparison of the parametric resonance AFM presented here and the conventional forced excited AFM, Figure 8 shows the amplitude–distance curves and connected responsivity and sensitivity curves as presented in Figure 5a. The yellow curves represent forced excited AFM, calculated with our previously published methods [4]. In contrast, the blue curves show the parametric resonance AFM. For both curves, the excitation parameters are selected to get an identical free amplitude, ${\hat{q}}_{0}\approx 2.7$ nm. Besides the excitation itself, the systems are completely identical. We see the qualitative difference of both modes in the amplitude–distance curves where in case of parametric resonance AFM an amplitude $\hat{q}=0$ nm is reached several nanometers before touching the sample surface, as explained in Figure 3e,f. Looking to the amplitude sensitivity, we see an improvement of 30% comparing the maxima of both curves. Additionally, for $\kappa_{\mathrm{ts}}>-6\cdot 10^{-4}$ the amplitude sensitivity of parametric resonance AFM is always larger compared to forced excited AFM. This is beneficial as our proposed mode is more robust against changes of operational regime. For amplitude sensitivity, this effect is even stronger. Throughout the range of tip–sample interaction (along $\kappa_{\mathrm{ts}}$), parametric resonance AFM gives lower, which means better sensitivity since the thermomechanical noise is also improved using parametric excitation. In this example, this improvement is about a factor of 4.5. The comparison of both minima, as the best sensitivity in each mode, gives an improvement by a factor of 5.7 when using parametric resonance AFM. Of course, this comparison shows one specific parameter set, but there is reason to assume that for larger free amplitudes the improvement could be even better (see Figure 5b). This needs to be proven separately, as the use of $\kappa_{\mathrm{ts}}$ for forced excited systems is only valid for small amplitudes [4,40].

## 4. Discussion

Regarding the choice of the nonlinear saturation function, the hyperbolic tangent, see (11), serves as educated guess. We believe that other functions, such as exponential functions with a similar shape, would achieve a similar result. Although the specific numerical result will change, we view this as an optimization problem, just like the specific choice of excitation parameters.

Another question may be how the amplitude noise develops during approach of the cantilever and sample surface to each other. This can be answered with Figure 3e–f. Here, it is shown that due to the tip–sample force during approach the parametric resonance is left. In combination with Figure 4b, it gets clear that amplitude noise should increase at the end of the approach as it increases, leaving parametric instability. This is demonstrated in Figure S1 in Supplementary Material where for an exemplary parameter set the amplitude noise is calculated during approach. It can be seen that the predicted effect occurs. Fortunately, the increase of amplitude noise starts at the very end of approach and therefore does not influence the calculations of Figure 5b where a constant amplitude noise along $\kappa_{\mathrm{ts}}$ is assumed.

Another future research objective could be whether our proposed excitation scheme also works for AFM operation in liquids where *Q* decreases significantly and therefore the threshold for parametric resonance rises. All parts that produce the parametric excitation, including the active cantilever itself, must be investigated whether to support higher gains. Of course, this is more of a technological than a physical issue.

Additionally, the robustness of the proposed approach needs to be investigated in future work to clarify how changes in, for example, temperature or the feedback circuit influence the system’s behavior.

## 5. Conclusions

To conclude, in this work we propose and develop a new excitation scheme for AM-AFM using nonlinear parametric excitation. We select a nonlinear limitation function $f_{\mathrm{nl}}=\tanh\left(\gamma |q|\right)$ to get the desired amplitude response behavior. In the following, we perform a numerical analysis resulting in amplitude response curves as well as further bifurcation diagrams showing how the excitation parameters *G*, γ, and η influence the system. Furthermore, we analyze the dynamic behavior of the AFM cantilever during approach to the sample by computing amplitude response curves for several tip–sample distances and find indication that parametric resonance AFM could prevent tip and sample damage and could be advantageous for non-contact operation by design. A stochastic simulation revealing the amplitude fluctuations due to thermomechanical noise for different excitation parameters is performed to provide an overview of how the excitation parameters influence the amplitude noise and with that the sensitivity of the parametric resonance AFM system. Our numerical findings are validated by experiments showing the amplitude response behavior, amplitude–distance curves of a parametrically excited AFM cantilever, and the amplitude noise. Lastly, we find that for a selected parameter set, there is an improvement in minimal sensitivity by a factor of 5.7 compared to an identical system under forced excitation. This improvement consists of an increase in responsivity of 30% and a decrease of thermomechanical noise by a factor of 4.5. To finalize, we see a promising opportunity to improve the sensitivity of an existing AFM system by simply changing the cantilever excitation from conventional forced excitation to parametric resonance while keeping the rest of the system untouched. This marks a novelty to existing works that investigated sensitivity of parametrically excited cantilevers using bifurcation detection. Besides that, the technological implementation into an existing commercial AFM should be feasible, as all relevant signals for the parametric feedback loop are measured in the AFM system anyway and the feedback hardware can be installed in between the existing signal chain. In future, the results could be transferred to other applications of resonant sensors.

## Data Availability

The raw data supporting the findings of this work is available from the corresponding author upon request. Additionally, we are working on publishing the raw data to be published in an open access repository.

## Supplementary Materials

The following supporting information can be downloaded at: https://www.mdpi.com/article/10.3390/s26154791/s1, Figure S1: Amplitude noise during approach of cantilever to sample surface.
