# Supplementary material for: Utilization of Nonlinear Parametric Resonance in Micro Sensor Probes to Enhance Atomic Force Microscope Resolution
Source: Sensors (Basel). 2026 Jul 28;26(15):4791. doi: 10.3390/s26154791 (PMC13469868; doi:10.3390/s26154791)
Supplement: Supplementary file 1 [file sensors-26-04791-s001.zip › sensors-4382579-supplementary.pdf]

Supplementary Material
Utilization of Nonlinear Parametric Resonance in Micro Sensor Probes to Enhance Atomic Force Microscope Resolution

Jonathan Ehrmann, Oliver Radler, Thomas Sattel

Mechatronics Group, Technische Universität Ilmenau, Max-Planck-Ring 12, 98693 Ilmenau, Thuringia, Germany

Correspondence: jonathan.ehrmann@tu-ilmenau.de; Tel.: +49 3677 691815

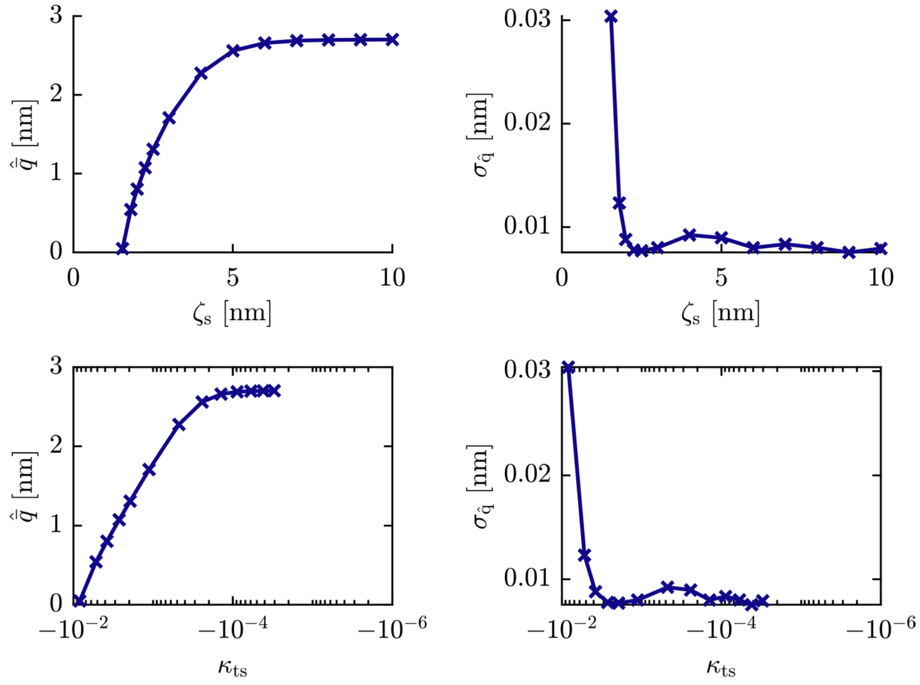

Figure S1: Amplitude noise during approach of cantilever to sample surface. Left column: mean of cantilever amplitude $\hat{\bar{q}}$. Right column: standard deviation of cantilever amplitude $\sigma_{\hat{q}}$. First row: influence of quasi-static tip-sample distance ζ_s on amplitude noise. Second row: Conversion of ζ_s to κ_{ts} to show influence of dimensionless tip-sample stiffness κ_{ts} on amplitude noise. Parametric excitation parameters are $G = 3.26, \gamma = 0.5, \eta = 2$.
